# Non-Canonical Inter-Protein Interactions of Key Proteins Belonging to Cytokinin Signaling Pathways

**DOI:** 10.3390/plants14101485

**Published:** 2025-05-15

**Authors:** Ekaterina M. Savelieva, Dmitry V. Arkhipov, Anna V. Kozinova, Georgy A. Romanov, Sergey N. Lomin

**Affiliations:** Timiryazev Institute of Plant Physiology of the Russian Academy of Sciences, Moscow 127276, Russia; savelievaek@ya.ru (E.M.S.); kozinovaav046@mgpu.ru (A.V.K.);

**Keywords:** cytokinin signaling, multistep phosphorelay, dimerization, signaling specificity

## Abstract

The multistep phosphorelay (MSP) is a conserved signaling system that allows plants to sense and respond to a variety of cues under rapidly changing environmental conditions. The MSP system comprises three main protein types: sensor histidine kinases, phosphotransmitters, and response regulators. There are numerous signaling pathways that use, in whole or in part, this set of proteins to transduce diverse signals. Among them, the cytokinin signal transduction system is the best-studied pathway, which utilizes the entire MSP cascade. Focusing on this system, we review here protein–protein interaction of MSP components that are not directly related to cytokinin signaling. These interactions are likely to play an essential role in hormonal crosstalk and may be promising targets for fine-tuning plant development. In addition, in light of recent advances in the study of cytokinin signaling, we discuss new insights into the putative molecular mechanisms that mediate the pleiotropic action of cytokinins and provide specificity for distinct MSP signals. A detailed network of known non-canonical protein–protein interactions related to cytokinin signaling was demonstrated.

## 1. Introduction

Cytokinins (CKs) are plant hormones involved in numerous processes of plant growth, development and stress responses. They regulate activity and size of shoot apical meristem [1,2], root meristem size [3], branching of shoots [4] and roots [5], cell division [6], chloroplast differentiation and leaf senescence [7], germination and flowering [8], and responses to biotic and abiotic stresses [9]. Such diversity and versatility of functions raise questions about mechanisms that allow one hormone to regulate so many processes throughout plant ontogenesis and how these mechanisms can be used for the benefit of agriculture. Despite the fact that the CK action in plants has been studied quite deeply, we still cannot fully control the CK system of plants, leaving only properties of these hormones useful for practical purposes. In this review, we attempt to consider protein–protein interaction of the CK signaling pathway members that are beyond the process of CK signal transduction in its “canonical” sense. We believe that an insight into this topic could help address the issue raised above.

In the commonly recognized “canonical” scheme, the CK signal transduction occurs via a multistep phosphorelay (MSP) system similar to the bacterial two-component system (TCS) [10]. It includes three types of key proteins: CK receptors (cytokinin histidine kinases, CHKs), phosphotransmitters (HPts) and response regulators (RRs). Signaling is initiated by CK free base binding to transmembrane (TM) CHK protein [11,12,13]. The activated cognate ligand receptor (in a dimer form) is autophosphorylated; the released “hot” phosphate is transferred from the CHK to the HPt, which carries it to the nucleus and phosphorylates the RR protein [14,15,16,17,18]. RR’s action is considered the final step of MSP [19]. Type-B RRs (RRBs), being the primary transcription factors (TF) of CK output, play a positive role in CK signaling, affecting target genes [14,16,20,21,22]. RRBs as TFs may act as dimers [23]. Type-A and type-C RRs (RRAs and RRCs, respectively) lack a DNA-binding motif and regulate CK signaling negatively [24,25,26,27].

Other negative regulators of CK signaling are pseudophosphotransmitters (PHPs), which are members of the HPt family, but they cannot be phosphorylated by histidine kinases (HKs) and therefore cannot phosphorylate RRs [28,29]. PHP of Arabidopsis (AHP6/PHP1) likely interacts with CK receptors to block HPt phosphotransfer activity [29]. For rice PHPs, they have been shown to interact directly with both CK receptors and RRs of different types [30,31].

Thus, in terms of CK signal transduction, we will call the protein–protein interaction in the scheme PHP—CHK ↔ CHK → HPt → RR(—RR)—PHP “canonical” in this review (the arrows indicate the direction of hot phosphate transfer, and the lines indicate an interaction not associated with phosphorylation). All other variants of protein–protein interaction involving these components (except for the interaction with CK metabolic enzymes) we will refer to as “non-canonical”. The examples discussed in this review show how non-canonical interactions increase the variability of CK output responses compared to what would be expected from the classical CK signaling scheme. Studying non-canonical protein–protein interaction of CK signaling components may provide insight into a wide range of issues both outside and inside the CK signaling system. On the one hand, these interactions are involved in crosstalk between different signaling pathways (e.g., ethylene, red light, osmosensing, auxin, gibberellin, and others); on the other hand, they may explain the high specificity of CK transcriptional responses and their seemingly paradoxical multidirectional action.

In this review, we attempted to cover all relevant studies that have demonstrated non-canonical interactions of key CK-related proteins over the nearly three decades since the current concept of CK signaling was formed. However, we have focused not only on the existing research on the topic but also highlighted relevant areas that remain either extremely understudied or not studied at all.

## 2. Cytokinin Receptors

The CK receptor is the first and key player in CK signal transduction. CK receptors are multidomain TM hybrid sensor histidine kinases. The extracytosolic part of the receptor is represented by a ligand-recognizing sensor module (SM), which consists of a dimerization interface region and a CHASE (Cyclase/Histidine kinase Associated SEnsory) domain composed of PAS and pseudo-PAS (PAS-like) subdomains [11,12,18,32]. SM is flanked on both sides by TM domains. The cytosolic part of the receptor includes the catalytic module formed of the HK-dimerization domain (HisKA or DHpD) and the H-ATPase (or CAD) domain [17,33]. And finally, the receiver-like (REC-like) domain and receiver domain (RD) form the receiver module at the C-terminus of the receptor [34].

In individual plant species, CK receptors are usually presented by a small family of proteins. For example, there are only three CK receptors in *Arabidopsis thaliana* [35,36,37] and the same number in monoploid potato *Solanum tuberosum* var. Phureja [38]. All members in such a family possess very similar structures but nevertheless differ in ligand specificity [39]. These properties arise from certain structural features that are quite important in the context of this review.

In general, the structure of CHKs is rather conserved. Two protein fragments that we can characterize as variable are the SM part responsible for ligand recognition and the REC-like domain. Meanwhile, most of the CHK protein molecule, including the HisKA domain, RD, and even another part of the SM (which forms the dimerization interface), are highly and, moreover, extremely conserved [12,17,18,33]. Variability of the SM part provides different ligand specificity of CK receptors, which is of great importance for long-distance signaling, when signals from a distant organ are more significant for the cell than signals from nearby tissues [39]. The functions of the REC-like domain (and the significance of its low conservation) remain poorly understood [18]. Among HKs, only CHKs have a REC-like domain [40]. It cannot participate in phosphotransfer and is tightly associated with the H-ATPase domain [41]. In summary, the complexity and high conservation of the structure of CK receptor proteins indicate their high specialization. All relatively variable regions are responsible for interaction with low-molecular ligands or intraprotein interdomain interactions. Such a structure apparently greatly limits the diversity of possible protein–protein interactions of CK receptors, leaving them almost 100% within the “canonical” scheme of CK signaling. In this review, we define canonical protein–protein interaction of CK receptors as their homo- and heterodimerization (with paralogs), as well as interaction with any member of the HPt family of the corresponding plant, which leads to phosphate transfer from HK to HPt or signal blocking with PHP. We found only a few pieces of evidence that CHKs might work in ways other than initiating CK signaling.

### 2.1. Negative Regulation of Cytokinin Signaling by Cytokinin Receptors

One way in which CK signaling is negatively regulated by receptor(s) is described by Mähönen et al. [42]. Although in this case the interactors are the CK and HPt, their interaction cannot be considered canonical due to the direction of phosphate transfer. As noted above, canonically it occurs downstream from the activated CHK to the HPt. However, inactivated CRE1/AHK4 (in the absence of the bound hormone) can remove phosphate from HPt. The authors suggest that other CK receptors may have similar phosphatase activity, but this has not been demonstrated experimentally. Remarkably, similar functions have been found in at least two other sensor HKs, which are, however, not CK receptors [43,44]. These studies are discussed in more detail below.

Recently a new negative feedback mechanism in CK signaling via CK receptor AHK4/CRE1 lacking an RD was discovered. This receptor is formed in plants by alternative splicing and has been shown to bind hormones and form dimers with full-length CK receptors. However, it is unable to activate the downstream signaling cascade [45]. Thus, canonical receptor interaction (dimerization) nevertheless results in a lack of further canonical interaction with HPt proteins and CK signal transduction.

### 2.2. Interaction of Cytokinin Receptors with Other Histidine Kinases

There is a suggestion that CHKs can form dimers with other HKs. One such HK is ETR1 (Ethylene Response 1), the first ethylene receptor identified in plants [46]. Ethylene receptors, as well as CK receptors, are believed to have a common ancestor protein and to have been acquired by plants from the cyanobacteria [16]. The ability of ETR1 to form heterodimers with CHKs has been proposed in the study by Ždárská et al. [47]. Several arguments can be made in favor of this theory. Within the ethylene receptor family, ETR1 exhibits promiscuity and is capable of forming heterodimers with any other ethylene receptor kinases [48]. ETR1 is localized in the ER membrane [48] like most of the CHKs [34,49,50]. Despite the available evidence for an ETR1-HPt interaction (discussed in detail below), the significant structural differences between the RD of ETR1 and the RD of CHKs [33] cast doubt on the ability of ETR1 to phosphorylate the conserved histidine (His) in the HPt phosphorylation site and mediate an ETR1-triggered phosphorelay [47]. Some evidence subsequently corroborated these doubts. It was shown that the RD of ETR1 is unable to accept the phosphate from the phosphorylated ETR1 HisKa domain [51]. Thus, the ETR1 phosphorelay signal is apparently realized through the formation of heterodimers with other HKs. The formation of such a heterodimer is shown for non-CK HK AHK5 [51,52].

It seems that the formation of a dimer between ETR1 and CHK may also involve ETR1 in MSP. However, there is no evidence for the existence of such a heterodimer even at the level of putative models. Perhaps a structural modeling of such a heterodimer would bring more clarity to this question.

### 2.3. Non-Canonical Interactions of Cytokinin Receptors with Other MSP Members

Another example of non-canonical interaction of the CK receptor was shown not directly in the plant, but using the yeast two-hybrid (Y2H) assay. In such a heterologous test system, interactions between the AHK2 receptor and the RRs (ARR2 and ARR14) were detected [53]. The authors note that for this interaction to make biological sense, the proteins must be located in the same subcellular compartment in plants. At that time localization of AHK2 was unknown. Nowadays, we know that AHK2 is localized predominantly on the endoplasmic reticulum (ER) membrane and, to a lesser extent, on the plasma membrane (PM) [34,49,50], while ARR2 and ARR14 as RRBs are localized in the nucleus [33,54,55,56]. The discovery of RRBs with non-nuclear localization seems unlikely because these types of RRs are TFs activating target genes [14,16,22]. Thus, it is doubtful that CK receptors can interact with RRs *in planta*. This interaction in the described experiment may be both an artifact of the method and an echo of the origin of MSP from bacterial TCSs.

### 2.4. Non-Canonical Cytokinin Receptor

Non-canonical CK receptor CHARK (CHASE domain receptor serine/threonine kinase) with serine/threonine (Ser/Thr) kinase activity is found in rice [57]. It is able to bind CKs and can function as a bona fide CK receptor. However, the protein–protein interaction by which it participates in CK signaling remains unknown. Dimerization of CHARK with canonical HK receptors is theoretically possible. For example, ETR1 is capable of forming heterodimers with serine/threonine ethylene receptor kinases [48,58]. However, the study of Halawa et al. [57] showed that even if such dimerization occurs, it is not the only way of involving CHARK in the CK signaling pathway.

The interaction of CHARK with HPts is possible in the case of receptors with dual (Ser/Thr and HK) activity. For example, for the ethylene receptors ERS1 and NTHK2, both HK and Ser/Thr kinase activity have been reported [59,60,61].

The interaction of CHARK with RRs can be assumed provided that RRs and/or non-canonical receptors undergo some posttranslational modification. Ser/Thr phosphorylation sites in RRs are unknown, but there are numerous posttranslational modifications of Ser/Thr residues in response to CKs in phosphoproteome [57,62]. Furthermore, CHARK has 4 TM domains, which casts doubt on its ability to interact with RRBs without being modified.

## 3. Phosphotransmitters

HPts, as well as CK receptors, are represented in plants by small families (five members in Arabidopsis, two members in rice) of single-domain proteins with histidine phosphotransfer activity [28,40,63]. In the canonical scheme of the CK signaling pathway, HPts are the downstream intermediate participants of signal transduction. They constantly cycle between the nucleus and the cytosol, bridging the membrane-bound receptors and RRs [33,64,65]. It should be noted that HPts can transfer phosphoryl groups not only into the nucleus but also to cytosolic-localized RRAs [65].

In more detail, functional HPts are able to accept the “hot” phosphate from a conserved aspartate (Asp) residue of the RD of the CK receptor to their own conserved His residue and then phosphorylate the conserved Asp residue of the RR protein [14,15,16,17]. HPts mostly (except AHP4 in Arabidopsis) act as positive regulators of the CK signaling pathway [28,64,66,67].

The structure of HPts is highly conserved [33], particularly in the area close to the catalytic His [68,69] and in the part forming the HK–HPt interaction interface [17]. HPts exhibit the promiscuity in interactions between CK receptors in different plants, both dicots and monocots [30,34,53,70,71], which is apparently explained by the high conservation of receptor RDs and HPt interaction interfaces [17]. Here, we consider the downstream transfer of phosphate in any CHK → HPt pairs within a single plant cell to be a canonical HPt interaction.

It is more difficult to clearly establish the boundaries of “canonicity” in relation to the interactions of HPt with RRs. For example, 23 RRs are known in Arabidopsis [70,71], and 28 are in the rice genome [72,73,74]. HPts can signal to different RRs of all types within a single cell [53,75]. However, there is no evidence that all RRs are involved in CK signal transduction [76]. Since HPts act as signaling hubs in the MSP system [77,78] and participate in a variety of direct interactions with proteins that are not involved in CK signaling (discussed in detail below), then a non-canonical HPt-RR interaction can exist. In this case, HPt must receive a signal not from the CK receptor while accepting RR must not be involved in CK signaling. However, at the moment we do not know of any such example. Thus, we regard any interaction in which HP phosphorylates RR as canonical.

Separately, it is worth considering pseudophosphotransmitters (PHPs), which are quite similar to HPts but lack the phosphoacceptor His replaced by another amino acid (aa) [28]. There is only one PHP in Arabidopsis [28] and 3 PHP in rice [30]. It is worth noting that functions of PHPs are not the same across plant species. In monocots, disruption of *PHP* genes results in a subset of phenotypes distinct from those of the analogous dicot mutants [79]. However, even in monocots, PHPs can act as negative regulators of CK signaling [31]. In this review, we will refer to the interaction of PHPs with both CK receptors and RRs as canonical.

### 3.1. Negative Regulation of Cytokinin Signaling by Phosphotransmitters

Using biolayer interferometry (BLI), it was shown that non-phosphorylated HPts of Arabidopsis (AHP1-5) could bind with RRB (ARR1), sequestering it. Thus, HPts may act as molecular sinks, temporarily inhibiting phosphotransfer [80]. Interestingly, in this case the HPt acts like PHP, which can also interact with RRs, apparently preventing their phosphorylation [30,31].

### 3.2. Dimerization of Phosphotransmitters

Dimerization of HPts falls into a gray area when defining (non)canonical interactions. This is rather surprising, since the ability of HPts to form dimers is not in doubt. The first evidence that HPts can form both homodimers (with the same protein) and heterodimers (with paralogs) was obtained by Y2H assay [53,55]. It was then shown that AHP2 forms homodimers in protoplasts [81]. Indirect evidence that AHP1 forms a homodimer was obtained in the work of Scharein and Groth [82]. Later, using bimolecular fluorescence complementation assays (BiFC), clear evidence that various HPts of Arabidopsis form homodimers in plant cells was obtained by Lomin et al. [34]. Recently, the formation of both homo- and heterodimers of apple (*Malus domestica*) HPts has been demonstrated by Y2H and BiFC assays [83]. In addition, a number of HPt homo- and heterodimer complexes (for AHP1-3 and StHP1) were modeled, and the properties of their dimerization interfaces have been described (including residues that probably determine the interactions) [17].

With this amount of evidence for HPt dimerization, its role in CK signal transduction is completely unclear. The HPt dimers were detected both in the nuclei of the plant cells [34,81] and in the cytosol [81]. However, there are no data on where they are formed: whether they can form in nuclei and get there from cytosol or vice versa. Accordingly, it is not known at what time point dimerization occurs: before, after, or during the acceptance of phosphate from the CK receptor.

It should be noted that in a recent study, Tran and Ruszkowski [80] have unexpectedly stated that “HPt proteins do not form homodimers”. The reason was the authors did not find a physiologically relevant HPt/HPt interface in different HPt crystal structures. This conclusion contradicts the former modeling of HPt structures by Arkhipov et al. [17]. This situation may lead to a dispute about the most appropriate approaches in relevant bioinformatics research. However, the conclusion of HPt dimerization in the study [80] contradicts existing experimental data. Nevertheless, when specifying the most likely monomeric status of HPts, authors often note that these are free HPts. Thus, the data they obtained do not exclude the possibility of dimer formation by phosphorylated HPts.

## 4. Interactions of Phosphotransmitters with Non-Cytokinin Histidine Kinases

As noted above, the structure of HPts is very conservative. Since all members of the HK family have a quite similar modular basic structure and the most differences in their architecture are found in their sensor domains [44], it is not surprising that HPts are able to interact not only with CK receptors but also with other HKs in the plants.

It was previously thought that the genome of the *Arabidopsis thaliana* contains 11 genes encoding HKs [40,84]. There are 3 CK receptors (discussed in detail above), 5 proteins involved in ethylene signaling pathway (ETR1, ERS1, ETR2, ERS2, and EIN4) [85,86,87] and 3 HKs (AHK1, CKI1, and AHK5/CKI2) lack the ability to perceive ethylene or cytokinin signals and have been attributed to a variety of plant processes such as osmoregulation and stomatal density control [88,89,90,91,92], stress responses [91,93,94], root growth [95,96], seed maturation [97] and megagametogenesis [98,99,100]. However, it was later established that among the kinases of the ethylene signaling pathway, only ETR1 has solely histidine kinase activity, whereas ETR2, ERS1, ERS2, and EIN4 have Ser/Thr or dual (Ser/Thr and HK) kinase activity [16,59,60,78,101]. In addition, these kinases have other significant differences from ETR1: ETR2, ERS2, and EIN4 have a degenerate catalytic domain; ERS1 and ERS2 miss a receiver domain at their C-terminus [44]. Thus, HPts can interact (in addition to CHKs) with ETR1, AHK1, CKI1, and AHK5/CKI2. We will discuss these interactions below.

### 4.1. Interactions of Phosphotransmitters with ETR1

Numerous studies have demonstrated that ETR1 physically interacts with HPt proteins: AHP1 [82,102,103], AHP2, AHP3 and AHP5 [47]. The interaction of ETR1 and AHP1 is characterized by a dissociation constant (K_d_) of 1.5 µM [103], which indicates the very high affinity of these two proteins. The affinity between ETR1 and AHP1 is altered by their phosphorylation state, where it is highest if one protein is phosphorylated and the other is not [82]. Moreover, there is evidence that HPts activated by ETR1 then transduce a signal to RRBs [104,105,106] and RRAs [47]. However, there are doubts about the ability of the ETR1 homodimer to transduce a signal to RRs. ETR1-initiated signaling appears to require other HKs (see above). Nevertheless, these results suggest the existence of crosstalk between CK and ethylene signaling pathways through direct interaction of ETR1 and HPt proteins.

### 4.2. Interactions of Phosphotransmitters with AHK1

AHK1 lacks the CK-binding part (CHASE domain) and therefore cannot be a CK receptor. However, like CK receptors, it can participate in the phosphotransfer process [88]. As shown with a Y2H and in vitro phosphorylation and phosphorelay assays, AHK1 is able to interact with AHP2 of Arabidopsis [44,102]. The K_d_ for the AHK1-AHP2 interaction is ∼300 nM, indicating the high stability of this phosphorelay complex [44].

The specific ligand for AHK1, if it exists, remains unknown so far. Nevertheless, it was established that ligand-less AHK1 may act as a phosphatase [44] like AHK4 [42] (detailed above). So AHK1 is capable of relaying “hot” phosphate from phosphorylated AHP2 on itself [44]. Thus, in this case, AHP2 is involved in a “double” non-canonical interaction, transferring phosphate upstream, not to the CK receptor.

In poplar (*Populus trichocarpa*), AHK1 orthologs are able to interact with 3 out of 10 corresponding HPts (HPt2, 7 and 9) [107,108]. Signal from AHK1 orthologs is transmitted via HPt to RRBs [107,108,109,110,111] and RRAs [97,112]. This suggests an interconnection between CK and osmosensing signaling pathways [113].

### 4.3. Interactions of Phosphotransmitters with CKI1

CKI1 (Cytokinin Independent 1) was identified as an activator of CK-like response and became a prospective candidate for a CK receptor [114]. But later it was shown that it does not bind CK molecules [115]. Although CKI1 is not actually a CK receptor, it is able to interact with almost all HPts of Arabidopsis. The interaction of CKI1 with AHP1 and AHP2 was first demonstrated in a Y2H assay [102]. And then Mähönen et al. [42] reported phosphotransfer occurs from CKI1 to the AHP1-3,5 in vitro. It has been proven that RD of CKI1 is necessary and sufficient for specific protein–protein interactions with HPts in *Arabidopsis* in vivo. At the same time, CKI1 interacts preferentially with AHP2 and AHP3, weakly with AHP5 and AHP1, and no interactions were detected with AHP4 and PHP1 (AHP6) [116]. CKI1 not only interacts with HPts but also shares downstream MSP signaling components with the CK signaling pathway. There is a genetic pathway consisting of CKI1, AHPs, RRBs and RRAs, in which CKI1 acts independently of CK receptor genes [100,117].

In addition, CKI1 acts as a phosphatase in vitro with phosphorylated AHP1 and AHP2 [43]. We discussed a similar function of AHK4 and AHK1 above.

### 4.4. Interactions of Phosphotransmitters with AHK5/CKI2

Initially, AHK5/CKI2, like CKI1, was considered a CK receptor [14,118], but was similarly later excluded from their ranks [119]. Among the HKs we are considering, AHK5 is unique in that it does not have a TM domain(s) and is localized not on a particular membrane of a cellular compartment but in the cytosol [119]. However, there is evidence that AHK5 is associated with the plasma membrane [89]. Similarly to other HKs from Arabidopsis, AHK5 is able to bind to a number of HPts. AHK5 forms a protein complex with AHP1-3,5 in Y2H assays [90]. Using the BiFC assay, only AHP1, 2, and 5 were first shown to interact [91]. However, co-expression of BiFC constructs in transformed tobacco then revealed the interaction of AHK5_RD_ with all members of the Arabidopsis HPt family except AHP4, but including the PHP (AHP6) with somewhat weaker affinity [120]. In surface plasmon resonance (SPR) experiments, AHK5 interacts with AHP1-3 with K_d_ values in the range of 2.7 to 4.4 µM [120]. Downstream signal from AHK5 and AHK5-interacting AHPs is taken up by RRAs (ARR4 and ARR7). The phosphorylation of ARR4 appears to be required for at least some of AHK5-dependent signal transduction to occur [90]. AHK5, AHP1-3,5, and ARR4,7 comprise their own signaling pathway [90], which they share with the CK pathway.

### 4.5. Interactions of Phosphotransmitters with CRFs

CRFs (cytokinin-response factors) are a subset of CK-regulated TFs whose phosphorylation causes them to bind DNA and activate transcription of CK-related genes. They are completely unrelated to the RRBs and have no overlapping domains with other CK signaling proteins [56]. Several reasons can be outlined why CRFs are not considered canonical members of the CK signaling pathway. Of the 12 CRFs found in Arabidopsis [121], only 8 of them (CRF1–CRF8) could directly interact with AHP1–AHP5 (except the interaction of AHP2 with CRF2 and CRF3) both in vitro and in vivo [122]. In addition to the CRFs that are phosphorylated by HPts, some CRFs interact with RRs [122,123,124,125]. CRFs regulate components of other hormonal pathways, they influence the auxin transport machinery, and they are likely involved in hormonal crosstalk [125,126,127,128]. Not all CRFs are CK-responsive [129], and diverse signals could induce the CRF expression, suggesting these proteins have roles far beyond response to their namesake phytohormone [125]. Thus, the known extensive interactions between CRFs and HPts make CRFs, if not canonical members of MSP, then a side branch of the CK signaling pathway [57,122].

### 4.6. Interactions of Phosphotransmitters with Molecular Switches

In rice, both of its HPts (OsHP1 and 2) directly interact with OsRAC3, a Rho GTPase-related molecular switch. OsRAC3 recruits OsHPs to the cytoplasm, inhibiting further signal transduction to the nucleus. OsRAC3 is activated by auxin; thus, the OsRAC3-OsHP1/2 auxin-induced interaction attenuates CK signaling and reveals molecular crosstalk between auxin and CK [130].

## 5. Response Regulators

In Arabidopsis, 32 genes of RRs have been identified [40], including 23 genes encoding proteins predicted to be functional RRs [77]. All of these 23 functional RRs contain in their RD a phosphoacceptor site consisting of conserved D-D-K residues, including a conserved Asp required for phosphotransfer from HPts [25]. The remaining nine genes encode pseudo-RRs (PRRs) that lack the conserved phosphorylatable Asp [131], while they share significant sequence similarity with the RD of RRs [24]. Some of PRRs contain a CCT motif, which plays a vital role in the regulation of circadian rhythms [26,74,132], so these proteins participate in modulating the circadian system [133,134]. Moreover, such PRRs operate in a CK-independent manner [131,132]; therefore, we will not consider them in this review. Four of the nine Arabidopsis PRR proteins both have no ability to undergo phosphorylation, and they are not circadian clock associated [77]. Nevertheless, no evidence could be found that they participate in CK signal transduction. Thus, we exclude all PRRs from further consideration.

All functional RRs can be categorized into distinct families: RRAs, RRBs and RRCs on the basis of their domain architecture, function and phylogenetic analysis [26,73,77].

*RRA* genes are rapidly induced by the CKs being primary response genes [135,136]. RRA proteins consist mainly of a receiver domain with small N- or/and C-terminal extensions [137]. There are 10 RRAs in Arabidopsis and 13 in rice [63,70,73]. They lack a DNA-binding motif and negatively regulate CK responses [14,24,25], acting as a negative feedback loop [25,70,138]. The mechanism of this negative regulation of CK signaling is not fully understood. It appears to involve both competition of RRAs with RRBs for “hot” phosphate and the interaction of RRAs with some regulatory proteins [25,76]. There is evidence that RRAs can directly form a complex with RRBs and thereby inactivate them [70]. Generally, RRAs localize in the nucleus, whereas several RRA proteins in Arabidopsis show subcellular localization in the cytoplasm [24,55,139,140].

The RRCs have a similar domain structure to the RRAs [26]. They also do not contain the DNA-binding domain and lack long C-terminal extensions. But RRCs are not phylogenetically closely related to the RRAs (nor are RRBs) [25,141]. The *RRC*s are not transcriptionally regulated by CKs, unlike *RRA*s [77,141]. However, they were shown to play a role in the CK signaling pathway [142]. Arabidopsis and rice each have two RRCs [26,73,77]. For at least one of the two Arabidopsis RRCs, there is evidence that it is working strictly within the phosphorelay as a phosphohistidine phosphatase and shows phosphocompetition with RRBs [27,143,144].

Positive regulators of CK signaling that have no repressive function are RRBs [128]. There are 11 RRBs in Arabidopsis and 13 in rice [25,73]. Phosphorylated RRBs are DNA-binding TFs mediating the transcriptional response to CKs [14,16,22,24,27,54,145]. These proteins differ from RRAs and RRCs in their more complex modular structure. Like other RRs, they have a receiver domain with a conserved Asp residue, which serves as the site for phosphorylation [146]. And they also have C-terminal extensions of variable length, containing a conserved Myb-like DNA-binding domain (GARP) followed by a putative glutamine-/proline-rich activation domain [23,147]. The Myb-like domain of the RRBs binds to a short core DNA sequence that is critical and sufficient for RRB binding [148]. The RD inhibits DNA binding by the RRB in its non-phosphorylated state. Phosphorylation of the conserved Asp relieves the inhibition and exposes the Myb-like DNA-binding motif, allowing the protein to bind to its targets and initiate transcription [149]. RRBs are necessary for both gene activation and repression in response to CKs [71,150].

Both in prokaryotes and eukaryotes, RRBs are capable of homodimerization for activation as TFs [10,22,151,152,153]. The formation of plant RRB homodimers was demonstrated with Y2H assay and *in planta* [53,111,154]. Crystal structure analysis of the RD-DBD complex (receiver domain-DNA binding domain) of plant RRB confirmed that they can homodimerize upon phosphorylation, and such a dimer can bind with DNA and promote transcription [155].

Unlike bacteria, for which there are almost no examples of physiologically significant heterodimerization of paralogous proteins [10,153], plant RRBs are also capable of heterodimerization. Moreover, cooperative action of different RRBs may be necessary for full transcriptional activation of particular target genes [156]. Heterodimerization of plant RRBs was confirmed with Y2H and BiFC assays [53,111,157]. For Arabidopsis ARR1 and rice Ehd1 (a close homolog of RRBs that has no orthologs in Arabidopsis [158], the dimerization regions have been defined [155,159]. In summary, dimerization of RRBs represents an essential component of CK response transcriptional regulation [23].

Thus, we classify homo- and heterodimerization of RRBs as canonical interactions. We also consider RRs phosphorylation with HPts and RRs interactions with PHP as canonical interactions. The other variants of RR’s interaction with other proteins are discussed as non-canonical in this review.

### 5.1. Dimerization of Type-A Response Regulators

In vivo dimer formation has been shown for RRAs. In particular, ARR5 strongly interacted with itself and formed homodimers/oligomers [160]. If dimerization of plant RRBs can be considered as a way to activate TFs, similar to bacterial RRs [10], then homodimerization of RRAs may be important for stability and interactions with working partners [160]. It should be noted there is a complete lack of information regarding the RRC’s dimerization.

### 5.2. Interactions of Response Regulators with Kinases

Above we reviewed the study that demonstrated the possibility of direct interaction of two Arabidopsis RRBs (ARR2 and ARR14) with the CK receptor AHK2 in the Y2H assay [53]. Above we discussed why such an interaction in a living plant cell seems unlikely. However, similar data are available for another sensor, HK, the ethylene receptor ETR1. It is able to biochemically bind to ARR2 and phosphorylate it in vitro [104]. Considering that ETR1, like AHK2, is a TM protein, similar doubts (detailed above) can be expressed here regarding the possibility of such interaction *in planta*. The authors of the study suggest the participation of HPt proteins in ARR2 modification.

There is experimental evidence that RRA (ARR5) can physically interact with Ser/Thr kinases SnRK2.2, SnRK2.3, and SnRK2.6, members of subgroup III SnRK2 (Sucrose non-fermenting-Related Kinase 2), and be phosphorylated by them in vitro and in vivo. RRBs (ARR1, ARR11 and ARR12) can also interact with these kinases in vivo, but their phosphorylation in this case is very weak. However, such interactions dramatically reduced the autophosphorylation activity of the kinase itself [160]. Since SnRK2 are the key kinases of the abscisic acid (ABA) signaling pathway, SnRK2-RR interactions appear to be the physical basis for cross-talk between ABA and CK signaling.

The RRCs are also worth mentioning here. Their sequences are more similar to the hybrid kinase RDs than to other RRs. This suggests that the HK protein, rather than HPt, may serve as their phosphodonor [77]. In addition, at least one RRC, ARR22, is a preferentially cytosolic protein [143]. This gives it the potential to interact with membrane-bound HKs, CK receptors in particular.

### 5.3. Interactions of Response Regulators with Other Transcriptional Factors and Transcriptional Modulators

The formation of TF complexes is a regulatory mechanism in prokaryotic and eukaryotic organisms that controls gene expression depending on input information [161]. Most of the information on direct RR-TF interactions concerns RRBs. On the one hand, RRBs function at the top of the transcriptional cascade, where they regulate subsequent waves of transcription via interactions with TFs. On the other hand, the activity of RRBs themselves can be regulated through interactions with TFs [76]. For a detailed insight into the interactions between RRBs and a wide range of TFs, we suggest referring to the review of Leuendorf and Schmülling [23].

In this review, we will provide only a few examples where RRBs in complex with other TFs or transcriptional modulators are found as an inducing (or inducible) component. Among the TFs and transcriptional modulators that regulate RRBs through direct interaction with them, we can highlight DELLA [157,162,163] and EIN3 [157] proteins. Complexes of ARR1 with DELLAs and EIN3 (as well as the formation of the ARR1-ARR12 heterodimer) enhance TF activity of ARR1 and appear to mediate crosstalk between CK and auxin, ethylene, and gibberellin signaling pathways [157]. Among the targets of RRBs, we can emphasize CRFs [122,123,124]. We have noted above that CRFs are able to be phosphorylated by HPts and are involved in CK-auxin crosstalk. In addition, some CRFs (CRF1, 2, and 6) can interact with RRBs (ARR10 and 12) as their downstream targets [57,122,128].

It is also necessary to note here the existence of complexes of RRBs with transcriptional repressors. TIE1/TIE2 can interact directly with a number of RRBs to repress transcription of its target genes [164,165].

There is much less data on the interaction of TFs with RRAs. ARR 7, like RRBs, interacts with CRFs, although the functions of CRF-RRA complexes remain unknown [122,128]. It has also been shown that a number of RRAs (ARR4-6) physically interact with the ABI5 (ABA-Insensitive 5, a key positive regulator of ABA responses) and suppress its transcriptional function [166]. RRAs may inhibit ABI5 from interacting with the proteasomal degradation machinery [167]. Such interactions may constitute the mechanism of hormone crosstalk.

RRA-TF complexes may participate in the CK feedback loop. A subset of RRAs (RRA4,5) interacts with BPCs (BASIC PENTACYSTEINES), TFs that induce a number of genes in response to CKs. RRAs are able to modulate (inhibit or stimulate) BPC activity depending on cell type and/or developmental stage. It is important to note that this interaction is independent of phosphorylation [168]. This interaction may (partly) explain the mechanism of negative regulation of CK signaling via RRAs.

### 5.4. Interactions of Response Regulators with Phytochromes

ARR3 and ARR4 (RRAs) can directly interact with phytochrome B (phyB) [169,170]. At least ARR4 stabilizes the phyB active form under the CK influence [167]. This implies a crosstalk between CK and light signaling [170,171].

### 5.5. Signal Overlap and Non-Canonical Interactions of CK-Related Proteins

Since the concept of CK signaling, which we can now call canonical, was formed, researchers have been asking the following same questions year after year:

(1) Why does the CK signaling system need such a redundancy of components? For example, there are 3/5 CHKs, 5/2 HPts, 10/13 RRAs, and 11/13 RRBs in Arabidopsis and rice, respectively. Such signal overlap seems unnecessary simply to create a failsafe system [56].
(2) How does a plant differentiate the source of a signal and form an adequate response? In other words, what is the basis for CK specificity? CKs affect a wide range of aspects of plant growth and development and regulate responses to biotic and abiotic stresses. The roles of these hormones can be different, and sometimes opposite, depending not only on the age of the plant and its stage of development but also on the type of organ and tissue where they work (reviewed, for example, in [8,172,173].

The fact that other signaling pathways use the same components of the CK signaling system complicates the answers to these questions. Therefore, the second question can be reformulated as follows: What is the molecular basis for different signaling pathway specificity in the MSP system?

Such question(s) are easier to answer from the end because, unlike signal transduction, the mechanism for realizing specific responses seems clearer. An adequate response to external signals is largely carried out through the interaction of RRs with proteins of different TF families. Indeed, TFs can act differently in certain cell types at certain periods of development, and this allows the pleiotropic effects of CKs to be realized [23,161,168]. Such complexes provide opportunities for crosstalk between signaling pathways. However, RRs may need not only to form complexes with different TFs but also to homo- or heterodimerize to function adequately. Moreover, it is important not only which dimers are able to form in the cell but also which dimers are impossible to form. In poplar, the separation of the CK and osmosensing pathways is partly due to the inability of specific RRBs to heterodimerize. It was shown the lack of interaction of RRBs involved in antagonistic developmental processes. So the specificity in RRBs dimerization is also the mechanism for avoiding unwanted signaling crosstalk [112].

Returning to the first part of the outlined questions, we have to admit that there is no clear understanding of what underlies the specificity of signal transduction in the MSP system. It is possible that some, but apparently non-primary, role in the isolation of a specific signal is played by the preferential localization of some signaling components in different plant organs and in different development stages. This can be called differential expression in space and time [119] or the cellular context [76]. A number of examples of such specifications can be given. In Arabidopsis, CK receptor AHK3 is expressed predominantly in aboveground organs, whereas AHK4 expression is highest in roots [1,174]. In potatoes, the genes of the *StHK3* and *StHK4* clades are expressed in roots in almost equal proportions, whereas expression of *StHK2* genes is relatively weak there [38]. Transcripts of all *AHPts* except *AHP4* are presented in roots, stems and leaves [28,66], while *AHP4* is predominantly expressed in flowers [175]. In apple, most of the 13 *MdAHP* family members are expressed in leaves, flowers and fruits, and only a minority are expressed in roots [83]. Some RRs also demonstrated different expression among organs and tissues. Two *RRBs* of Arabidopsis (*ARR2* and *ARR18)* have been shown to be expressed specifically in the anthers [147,176]. One of two Arabidopsis *RRCs*, *ARR22*, is expressed in flower tissues [141,177] and buds [175], whereas the second *RRC*, *ARR24,* is primarily expressed in pollen grains. Spatial patterns of *ARR22* and *ARR24* expressions do not overlap [177]. It may also be noted that different expression in tissues and organs of some HKs with phosphatase activity [42,43,44] may also provide some diversity in signal transduction via different phosphoload in the cells [42].

However, localization exclusively in one particular organ or expression at one particular stage of development is not typical for MSP components. There are many proteins whose expression is at approximately the same level in several plant organs, both above- and underground, throughout a significant part of ontogenesis. For example, expression of AHP3 and AHP5 is ubiquitous in leaves, stems and roots of Arabidopsis [178]. CK receptor gene *AHK2* is expressed to about the same degree in leaves and roots [174]. The transcripts of *ARR1* and *ARR11* genes are detected in Arabidopsis in roots, leaves, stems, flowers, and siliques in plants of different ages [179]. In addition, preferential localization in a certain organ(s) does not mean its complete absence in others. Thus, “root” CK receptor AHK4 is expressed in aboveground organs, but to a lesser extent.

Currently, no unique combination of MSP members is known that creates a separate chain from signal-initiating components to TFs. Hypotheses that the specificity of signal transduction is accomplished by covalent modification of the proteins involved [77] or by unique scaffold or chaperone-like proteins that can separate subsets of proteins into distinct signaling complexes [56,77,119] require the recruitment of a vast multitude of modifiers. To date, we are not aware of any such examples in the context under consideration. However, it is possible that the separation of signals occurs not due to a unique combination but due to a more or less preferable combination of different signal transduction elements.

Although HPts, which have been repeatedly referred to as signaling hubs in numerous studies, are characterized by high promiscuity with upstream and downstream components [17,34,53], their promiscuity is not absolute. In the Y2H assay, the rice CK receptor OsHK4 was shown to interact with only one of the two HPt (OsHP2) [30]. CKI1 does not interact with AHP4 and AHP6 [116], and AHK5 does not interact with AHP4 [120]. In poplar, AHK1 orthologs are able to interact with 3 out of 10 corresponding HPts (HPt2, 7 and 9). In this case, there are HPts that are common to both HK1 and CHKs, and there are those that are specific to CHKs. Thus, crosstalk can occur from CK to the osmosensing pathway, but not vice versa [113]

However, attempting to explain the separation of signaling pathways through such selectivity seems unsatisfactory in a number of cases. For example, a non-CK protein, AHK5/CKI2, binds AHP1-3 with similar values as CK receptors AHK2-4 do [17,120]. Moreover, it seems that this explanation is completely inapplicable for rice with its two HPts. It has been shown that rice HPts interact with multiple histidine kinases, and different signaling pathways can share the same HPts [30]. In addition, such examples of selectivity cannot explain CK specificity, since CK receptors interact with HPts with approximately the same affinity [17,30].

In summary, regarding the question of signal transduction specificity in MSP, none of the available hypotheses (nor any combination of them) seem fully convincing. Intriguingly, the gap in our knowledge of signal transduction specificity coincides with the lack of understanding of the role of dimerization of HPts, which are directly involved in signal transduction. The question arises whether HPts dimerization could play a role in the realization of signal transduction specificity in the MSP system. Despite the paucity of studies of HPt dimerization (and the uncomplete data on other MSP members), there are some arguments in favor of the involvement of HPt dimers in the process of signaling pathway specification.

It has been shown that the ETR1-AHK5 heterodimer mediates ethylene-initiated and CK-independent hormonal control of root growth. In such a dimer, initiation of signaling by ethylene leads to transphosphorylation: HisKa of ETR1 phosphorylates AHK5_RD_ to initiate a phosphorelay. Thus, the phosphate from ETR1 transfers through the RD of AHK5 and subsequently to HPt, independently of the HK activity of AHK5 [51]. This example shows, that the signal-transducing HPt must “know” not only the composition of the HK dimer from which it takes up phosphate but also which receptor subunit has been autophosphorylated. It seems very unlikely that such specificity of the signal can arise during the movement of already phosphorylated HPt from the cytosol to the nucleus. So it should be determined by the HK initiating the signal.

It is possible that the promiscuity of HPts to HKs, observed in different experiments, is not so high in plant cells due to the fact that *in planta* HPts have to interact with HK dimers, both homo- and hetero-. In addition, both the composition of the kinase dimer and the position of the phosphate on one or the other of its RDs may influence its affinity to HPts. Nevertheless, the possible increase in the HPts selectivity to HKs *in planta* compared to in vitro and in silico data alone cannot explain the apparent specificity in signal transduction, especially in plants with a small amount of HPt variants, such as rice. However, the formation of specific HPt dimers simultaneously with or immediately after the acceptance of phosphate from HKs could significantly separate the signals coming from different sources. HKs can form nearby local increases in the concentration of dissociated free HPt monomers, the composition of which depends on their affinity for a particular receptor subunit. Thus, phosphorylated HPt is highly likely to form a specific pair with another HPt almost immediately after phosphate acceptance. It is also conceivable that HPt can attach not only to the phosphorylated RD but also to the RD of the second subunit of the kinase dimer. For example, the possibility of attachment of unphosphorylated HPt to the RD of RR, which is quite similar to the RD of HKs and contains a conserved Asp, has been shown [80]. The involvement of PHPs in the formation of dimers with HPts can also be assumed. PHPs and HPts are proteins of the same family, differing in the presence or absence of a conserved His in the phosphorylation site. This difference does not exclude the possibility of the formation of HPt-PHP dimers that can transduce phosphate.

Available data confirm that HPt dimer formation occurs after phosphate acceptance. The dimerization interface of HPt partially overlaps with the interface of interaction with HK_RD_. In addition, it was shown that trends in HPt-HPt interaction are somewhat ambiguous, and the HK–HPt complex seems to be less stable than the HPt-HPt dimer [17]. This suggests that in the dimerized state, HPts does not appear to be able to accept phosphate from the HKs. And the possible formation of dimers before phosphate acceptance would apparently reduce the efficiency of signaling. This conclusion is in accordance with the data that free HPts are most likely monomeric [80]. Movement of the phosphorylated dimer into the nucleus is also possible since the size of the HPt dimer is near the size of the nuclear pores [81]. It may also be noted that the phosphorylated dimer, unlike the monomer, is protected from phosphate loss and dephosphorylation by kinases with phosphatase activity.

In the nucleus, the HPt dimer is expected to specifically transfer phosphate to the correct RR or RR dimer/RR-TF complex. At this point, it is difficult to say what might trigger the specific decomposition of the HPt dimer and subsequent phosphorylation of the RR. In general, the mechanisms of the RR phosphorylation process (whether by monomer or dimer of HPt) raise questions. The conformation of the HPt complex with RR’s RD obtained using molecular modeling is similar to a complex with HK’s RD [17]. According to the existing models, HPt, having accepted phosphate from the Asp of HK_RD_, is repelled from it. In such a case, it is not clear what makes phosphorylated HPt (monomer or dimer) bind to RD of RRs and transfer phosphate to its Asp. Considering how the interactions of HPts-RRs are still poorly understood, it is almost impossible to both construct a consistent theory of the specific interaction of HPt dimers with RRs and find arguments against it.

It is important to note all or almost all known plant HKs form homodimers. AHK1-4, CKI1, and ETR1 of Arabidopsis, as well as HK1 of poplar, show such ability [34,44,53,108,117,180,181,182]. Probably, AHK5/CKI2 is also organized as a homodimer, but there is no direct experimental confirmation of this. However, AHK5/CKI2 (and orthologs) forms heterodimers with ethylene receptors [51,52]. Heterodimerization is also found in CK receptors (with transphosphorylation between HisKa domains) [183]. In ethylene receptors, their different combinations as homo- or heterodimers provide different output signals [184,185]. It is likely that, in general, the various kinase dimerization variants serve a similar function. Thus, it is conceivable that the two subunits of the kinase dimer may determine the subsequent dimerization of HPts by attaching (or excluding the attachment of) certain phosphotransfer proteins.

In summary, the assumption that HPt dimers may mediate signal specification in MSP seems attractive because it avoids “multiplication of entities” in the form of additional modifying factors or scaffold proteins. In any case, whether this assumption turns out to be correct or not, we hope that this discussion has highlighted how many questions still need to be answered about the functioning of the MSP system. In particular, studying the biological role of HPts dimerization is necessary for a better understanding of signal transduction processes in the plant cell.

## 6. Conclusions and Perspectives

**Figure 1:**
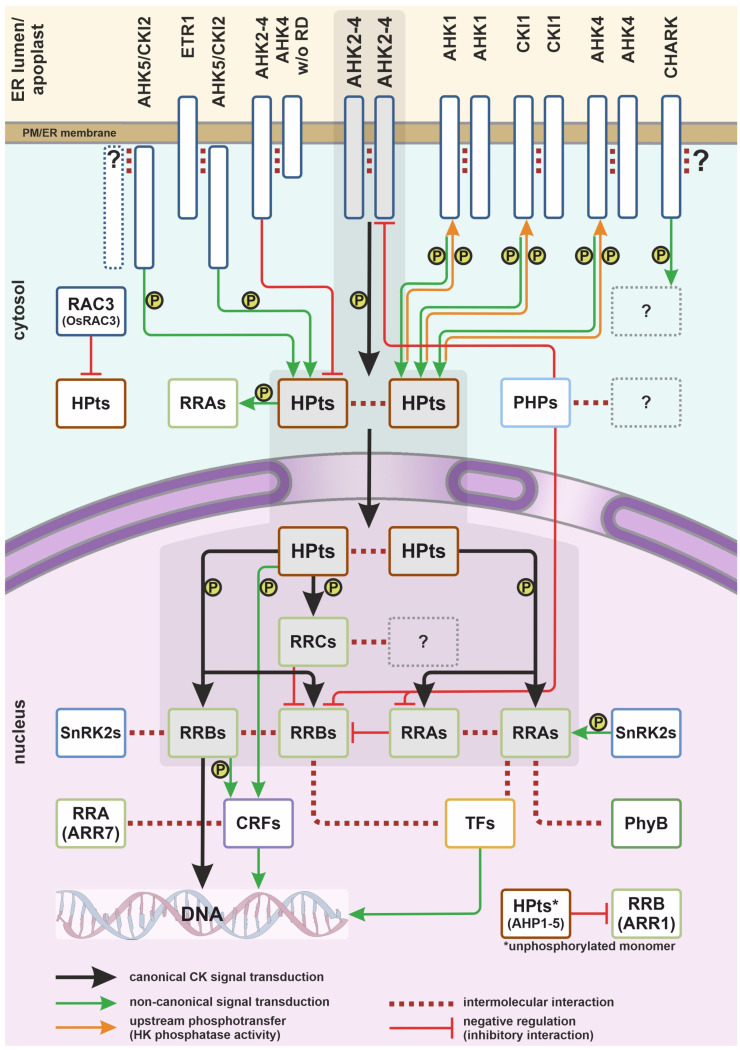
Integrative scheme of multistep phosphorelay in plants. AHK—Arabidopsis Histidine Kinase; AHK5/CKI2—Arabidopsis Histidine Kinase 5/Cytokinin Independent 2; CHARK-CHASE domain receptor serine/threonine kinase, cytokinin receptor; CKI1—Cytokinin Independent 1, histidine kinase; CRFs—Cytokinin Response Factors; ER—endoplasmic reticulum; ETR1—Ethylene Response 1, ethylene receptor, histidine kinase; HPts—phosphotransfer proteins; PHPs—pseudo-phosphotransfer proteins; PhyB—phytochrome B; OsRAC3—GTP binding protein of GTPase Rac/Rop family; RRA—type-A response regulator; RRB—type-B response regulator; RRC—type-C response regulator; SnRK2s—Sucrose non-fermenting-1-Related Protein Kinases 2; TFs—transcription factors; w/o RD—without receiver domain. Active phosphate is labeled as “P” in the circle. Shaded area—canonical CK signaling pathway.

In this review, we attempted to explain the pleiotropic role and multidirectional action of CKs by going beyond the canonical scheme of CK signal transduction at the level of protein–protein interaction (except for protein degradation issues). Almost all non-canonical interactions of the CK signaling pathway members may be promising targets for fine-tuning various processes in plants. Most interactions between CK and non-CK proteins are the molecular basis for crosstalk with other signaling pathways, and their regulation may certainly be of particular interest in terms of improving agricultural productivity (Table 1 and Figure 1).

However, the understanding of the molecular basis of such interactions is currently unsatisfactory. Moreover, going beyond the existing classical CK signaling scheme shows how many knowledge gaps still remain with respect to the chain of canonical CK interactions. In order to fully answer questions concerning the overall operation of MSP (e.g., the specificity of signal transduction), many specific questions should first be answered. Notably, many of them have remained unresolved for decades. This state of affairs may be, among other things, that the prospects for their study are underestimated. An example is the study of PHPs. The interaction of Arabidopsis PHP (AHP6) with CHKs is characterized as probable [29], but has not been shown by direct evidence. Only more than a decade later, in another plant (rice), it was shown that PHPs interact directly with CK receptors. Moreover, rice PHPs also interact with RRs [30]. Given that PHPs act in monocots and dicots in ways that are not quite the same [31,79,186], it remains unknown whether the protein–protein interaction of rice PHPs and AHP6 corresponds. If AHP6 interacts directly with CK receptors, does it interact with all of them or only with some? Does AHP6 interact with ARRs, and if so, with which ones? Can PHPs form homodimers like functional HPts? Can PHPs form heterodimers with HPts? The answers to these questions could significantly complement and perhaps change the classical scheme of CK signaling.

The model plant used for research can influence the determination of the relevance of studying specific questions. Conducting the vast majority of MSP studies on Arabidopsis introduces certain distortions into the interpretation of the results obtained. For example, the data and conclusions about the functioning of families of ten or more HPts in apple and poplar [83,107,108] can be extrapolated to some extent to Arabidopsis but can hardly be applied to explain the mechanism of functioning of only two HPts in rice. It is also difficult to explain the predominance of PHPs over HPts (three and two paralogs, respectively) in rice based on the Arabidopsis model (one and five paralogs, respectively). Nothing is known about the protein–protein interaction of the non-canonical rice CHARK receptor, which has no orthologs in Arabidopsis [57].

But even in the interactions of canonical CK proteins of Arabidopsis, there is much that is unclear. A number of questions remain unanswered regarding receptor proteins (detailed in [18]), although this is the most studied component of the CK signaling pathway. But for RRs, there are not even models describing their phosphorylation with HPts. Interaction relationships for several RRs were not studied at all [76,187]. And, of course, the importance of dimerization of MSP components in plants seems to be greatly underestimated. The role of HPts dimerization needs to be established.

Without answers to these and other questions concerning the transduction of the CK signal, it is impossible to fully understand the mechanism of MSP functioning and non-CK signaling pathways that use it. However, a look at the CK signaling system “from the outside” can allow us to change the usual focus on some details and emphasize the importance of solving underestimated problems.

## Figures and Tables

**Table 1:** Non-canonical interactions of CK-related proteins and their role in plants.

CK-Related Protein	Interaction Details	Interaction Partner	Role of Interaction	Experimental Evidence (Assay, Method)	References
AHK2-4	heterodimerization	CRE1^int7^ (AHK4 w/o RD)	Negative regulation of CK signaling.	BiFC	[44]
AHK4	← phosphate	AHP1-3,5	Negative regulation of CK signaling.	Phosphotransfer in vitro, yeast system	[41]
AHK2	phosphate →	ARR12,14 (type-B),	Unknown.	Y2H	[52]
AHP1-5	complex formation in the absence of phosphate	ARR1	Negative regulation of CK signaling.	BLI	[79]
Unknown	complex formation	CHARK	Branch of the cytokinin signaling pathway.	No	[56]
AHP2-3,5	homo- and heterodimerization	AHP5	Unknown.	Y2H	[52]
AHP2	homodimerization	AHP2	Unknown.	BiFC	[80]
AHP1	homodimerization	AHP1	Unknown.	Protein electrophoresis in vitro	[81]
AHP1-3	homodimerization	AHP1-3	Unknown.	BiFC	[33]
MdAHP1,6	heterodimerization	MdAHP3	Regulation of adventitious root formation.	BiFC Y2H	[83]
AHP1-3, StHP1a	homodimerization	AHP1-3, StHP1a	Unknown.	In silico methods	[17]
AHP1-3,5	← phosphate	ETR1	Crosstalk between CK and ethylene signaling pathways.	BiFC Y2H	[46,81,102,103]
AHP2 HPt2,7 and 9 of poplar	← phosphate	AHK1 (and orthologs)	Positive regulation of AHK1 signaling Crosstalk between CK, osmosensing and ABA signaling pathways.	Microscale thermophoresis in vitro, Y2H BiFC	[44,102,107]
AHP2	phosphate →	AHK1	Negative regulation of AHK1 signaling.	Microscale thermophoresis in vitro	[43]
AHP1,2,3,5	← phosphate	CKI1	Positive regulation of CKI1 signaling.	Y2H, BiFC, Microscale thermophoresis in vitro	[41,100,102,116,117]
AHP1,2	phosphate →	CKI1	Negative regulation of CKI1 signaling.	Phosphotransfer in vitro	[43]
AHP1-3,5,6	← phosphate	AHK5/CKI2	Positive regulation of AHK5 signaling. Crosstalk between CK, ethylene and ABA signaling pathways.	Y2H, BiFC, SPR	[89,90,95,120]
AHP1-5	phosphate →	CRF1-8	Branch of the cytokinin signaling pathway. Crosstalk between CK and auxin signaling (for CRF2,3,6).	Y2H, BiFC	[122,124]
ARR10,12 (RRBs) ARR7 (RRA)	complex formation	CRF1,2,6			
OsHP1,2	complex formation	OsRAC3	Inhibition of CK signal transduction. Crosstalk between CK and auxin signaling pathways.	Y2H Pull down assay and Co-IP in vitro	[130]
ARR5 (RRA)	homodimerization	ARR5	Maintenance of the protein stability. Interaction with working partners.	Co-IP in vitro, LCI	[160]
ARR2 (RRB)	← phosphate	ETR1	Crosstalk between CK and ethylene signaling pathways.	Phosphotransfer in vitro	[104]
ARR5 (RRA) 1,11,12 (RRBs)	← phosphate	SnRK2.2, SnRK2.3, SnRK2.6	Crosstalk between CK and ABA signaling pathways. Maintenance of the ARR5 stability. Suppressing the activity of the kinases (for RRBs).	BiFC, LCI	[160]
ARR1 (RRB)	complex formation	DELLAs	Increase in the ARR1 transcriptional activity. Crosstalk between CK, auxin and gibberellin signaling pathways.	Co-IP in vitro, Y2H	[155,162,163]
ARR1 (RRB)	complex formation	EIN3	Increase in the ARR1 transcriptional activity. Crosstalk between CK and ethylene signaling pathways.	BiFC	[155]
ARR4-6 (RRAs)	complex formation	ABI5	Crosstalk between CK and ABA signaling pathways.	Pull down assay in vitro, Y2H, BiFC	[166,167]
ARR4,5 (RRAs)	complex formation	BPC1,6	Regulation (positive/negative) of CK signaling.	Y2H	[168]
ARR11 (RRB)	complex formation	TIE1	Transcriptional suppression of CK target gene(s).	Y2H	[164,165]
ARR1,2,10,11,12,14,18 (RRBs)	complex formation	TIE2	Transcriptional suppression of CK target gene(s).	Y2H, LCI, Co-IP in vitro	
ARR3, ARR4 (RRAs)	complex formation	phyB	PhyB active form stabilization. Positive regulation of red light signaling. Crosstalk between CK and red light signaling.	Pull down assay in vitro, Y2H	[169,170]

← phosphate, phosphate →—direction of phosphate transfer during protein interactions. Co-IP—co-immunoprecipitation assay. LCI—luciferase complementation imaging assay. Y2H—yeast two-hybrid assay. BiFC—bimolecular fluorescence complementation assay. BLI—bio-layer interferometry assay.
